# Predictors of patient-reported quality of care in low- and middle-income countries: a four-country survey of person-centered care

**DOI:** 10.1093/intqhc/mzab110

**Published:** 2021-07-28

**Authors:** June-Ho Kim, Griffith A Bell, Hannah L Ratcliffe, Leah Moncada, Stuart Lipsitz, Lisa R Hirschhorn, Asaf Bitton, Dan Schwarz

**Affiliations:** Ariadne Labs, Brigham and Women’s Hospital & Harvard T.H. Chan School of Public Health, 401 Park Drive, 3rd Floor East, Boston, MA 02215, USA; Harvard Medical School, 25 Shattuck Street, Boston, MA 02115, USA; Division of General Internal Medicine and Primary Care, Department of Medicine, Brigham and Women’s Hospital, 75 Francis St, Boston, MA 02115, USA; Ariadne Labs, Brigham and Women’s Hospital & Harvard T.H. Chan School of Public Health, 401 Park Drive, 3rd Floor East, Boston, MA 02215, USA; Ariadne Labs, Brigham and Women’s Hospital & Harvard T.H. Chan School of Public Health, 401 Park Drive, 3rd Floor East, Boston, MA 02215, USA; RIWI Corp, 180 Bloor Street West, Suite 1000, Toronto, ON M5S 2V6, Canada; Ariadne Labs, Brigham and Women’s Hospital & Harvard T.H. Chan School of Public Health, 401 Park Drive, 3rd Floor East, Boston, MA 02215, USA; Center for Surgery and Public Health, Brigham and Women’s Hospital, 75 Francis St, Boston, MA 02115, USA; Ariadne Labs, Brigham and Women’s Hospital & Harvard T.H. Chan School of Public Health, 401 Park Drive, 3rd Floor East, Boston, MA 02215, USA; Department of Medical Social Sciences, Northwestern University Feinberg School of Medicine, 420 E Superior St, Chicago, IL 60611, USA; Ariadne Labs, Brigham and Women’s Hospital & Harvard T.H. Chan School of Public Health, 401 Park Drive, 3rd Floor East, Boston, MA 02215, USA; Harvard Medical School, 25 Shattuck Street, Boston, MA 02115, USA; Division of General Internal Medicine and Primary Care, Department of Medicine, Brigham and Women’s Hospital, 75 Francis St, Boston, MA 02115, USA; Ariadne Labs, Brigham and Women’s Hospital & Harvard T.H. Chan School of Public Health, 401 Park Drive, 3rd Floor East, Boston, MA 02215, USA; Harvard Medical School, 25 Shattuck Street, Boston, MA 02115, USA; Division of Global Health Equity, Department of Medicine, Brigham and Women’s Hospital, 75 Francis St, Boston, MA 02115, USA

**Keywords:** patient-centered care, patient experience, patient satisfaction, quality measurement

## Abstract

**Background:**

Person-centeredness is a foundation of high-quality health systems but is poorly measured in low- and middle-income countries (LMICs). We piloted an online survey of four LMICs to identify the prevalence and correlates of excellent patient-reported quality of care (QOC).

**Objective:**

The aims of this study were to investigate the examine people’s overall ratings of care quality in relation to their experiences seeking care in their respective health systems as well as individual-, provider- and facility-level predictors.

**Methods:**

We administered a cross-sectional online survey using Random Domain Intercept Technology to collect a sample of random internet users across India, Kenya, Mexico and Nigeria in November 2016. The primary outcome was patient-reported QOC. Covariates included age, gender, level of education, urban/rural residence, person for whom care was sought, type of provider seen, public or private sector status of the health facility and type of facility. The exposure was an index of health system responsiveness based on a framework from the World Health Organization. We used descriptive statistics to determine the prevalence of excellent patient-reported QOC and multivariable Poisson regression to calculate adjusted prevalence ratios (aPRs) for predictors of excellent patient-reported quality.

**Results:**

Fourteen thousand and eight people completed the survey (22.6% completion rate). Survey respondents tended to be young, male, well-educated and urban-dwelling, reflective of the demographic of the internet-using population. Four thousand one and ninety-one (29.9%) respondents sought care in the prior 6 months. Of those, 21.8% rated their QOC as excellent. The highest proportion of respondents gave the top rating for wait time (44.6%), while the lowest proportion gave the top rating for facility cleanliness (21.7%). In an adjusted analysis, people who experienced the highest level of health system responsiveness were significantly more likely to report excellent QOC compared to those who did not (aPR 8.61, 95% confidence interval [95% CI]: 7.50, 9.89). In the adjusted model, urban-dwelling individuals were less likely to report excellent quality compared to rural-dwelling individuals (aPR 0.88, 95% CI: 0.78, 0.99). People who saw community health workers (aPR 1.37, 95% CI: 1.12, 1.67) and specialists (aPR 1.30, 95% CI: 1.12, 1.50) were more likely to report excellent quality than those who saw primary care providers. High perceived respect from the provider or staff was most highly associated with excellent ratings of quality, while ratings of wait time corresponded the least.

**Conclusion:**

Patient-reported QOC is low in four LMICs, even among a well-educated, young population of internet users. Better health system responsiveness may be associated with better ratings of care quality. Improving person-centered care will be an important component of building high-quality health systems in these LMICs.

## Introduction

Rising expectations of the person-centeredness of health care, variable health outcomes and evolving disease priorities in low- and middle-income countries (LMICs) have highlighted the need for high-quality health systems [1]. With countries committing to achieve universal health coverage by 2030, there is growing recognition that access to services is not enough unless that care is effective, safe, trusted and person-centered [2].

High-quality health systems are person-centered, equitable, resilient and efficient [1]. In their 2018 report, the National Academies of Sciences, Engineering, and Medicine defined person-centeredness as ‘respectful of and responsive to individual preferences, needs, and values’ [3]. Within person-centeredness, measurement of the concept includes patient experience, a process measure and patient satisfaction, an outcome measure [1, 4]. Positive patient experience carries intrinsic value and can also lead to better health care outcomes, including patient satisfaction [5, 6]. To operationalize the concept of patient experience, the World Health Organization (WHO) designed a framework of ‘health system responsiveness’—the actual experience of people’s interaction with their health system in relation to their legitimate expectations [7].

Broadly, there is evidence that poor person-centeredness is widespread in LMICs, from disrespect of women during childbirth to distrust of response teams amid epidemics [8–10]. In a recent study across 12 LMICs, only 32% of individuals reported being very confident that they could receive effective care if they were to become very sick [10].

Despite its importance, person-centeredness is poorly measured in LMICs and rarely in a systematic and structured manner that truly represents the views of the end user [4]. However, the growth of mobile and internet technology around the world allows for more rapid and large-scale surveying and data collection of populations [10].

In this study, we piloted an online survey of four LMICs—India, Kenya, Mexico and Nigeria—to examine people’s overall ratings of care quality, representing the outcome of patient satisfaction, in relation to their experiences seeking care in their respective health systems, as measured by the WHO domains of health system responsiveness, as well as individual-, provider- and facility-level predictors.

## Methods

### Design

Our study was based on a cross-sectional, online survey designed by the study team. Questions on health system responsiveness were based on the WHO’s World Health Survey Responsiveness Module, which were also used by the study team in surveys conducted through the Performance Monitoring for Action platform [4, 7].

The survey was administered via Random Domain Intercept Technology (RDIT), a method developed by RIWI Corp (https://www.riwi.com; Toronto, Canada) and utilized in prior evaluations of health-care delivery [1, 11]. RIWI randomly delivers anonymous opt-in surveys to internet users and optimizes the survey to anticipate a range of characteristics, with the goal of making the survey accessible to the broadest scope of the internet population [12]. Ultimately, the resulting unweighted sample is representative of the country’s internet-using population [10, 11].

### Measures

Respondents reported their demographic characteristics including age, gender, level of education and urban/rural residence. Those who reported having sought any type of care in the past 6 months also reported information about the person for whom care was sought if they were seeking care for someone other than themselves. They also reported the type of provider seen, public or private sector status of the facility and type of facility. These independent variables were factors that could be hypothesized to be a predictor of a patient’s health-care experience [4, 13].

To assess patient experience, the survey measured different dimensions of health system responsiveness based on the framework established by the WHO [7]: dignity; quality of basic amenities, surroundings and environment; prompt attention and communication. Each question was assessed using a 5-point Likert scale rating, except for communication, which was measured using a 4-point scale (Supplementary Material 1).

The primary outcome of interest was a measure of patient satisfaction: the respondents’ rating of the overall quality of care (QOC).

### Participants, setting and data collection

Our survey was fielded over a prespecified 2-week period on 17–29 November 2016 in India, Mexico, Kenya and Nigeria. These four countries were selected for their low and middle-income status and having internet penetration levels above 20%. In 2016, the percentages of internet use in the population in India, Mexico, Kenya and Nigeria were 34.8%, 45.1%, 45.0% and 46.1%, respectively [14]. Surveys were administered in English except in Mexico where they were administered in Spanish.

### Sample size considerations

The survey was closed when at least 1000 surveys were completed in each country to achieve a margin of error of ±3% at a 95% confidence level (95% CI) in the four countries. The survey completion rate was 22.6%, with a resulting sample of 14 008 people out of 61 982 people who opted in to the survey, which was similar to the range found in past online surveys (8.5–31%) [11].

### Data analysis

For our analysis, Likert scale responses were re-scaled to a scale of 0–1. We created a ‘responsiveness index’ as a summary score using a mean of the four scores of questions about health system responsiveness, adapted from a prior index [4]. After compilation, the index was partitioned into five quintiles for ease of interpretation and comparability to previous literature.

Next, we compared unadjusted counts and proportions of respondents who rated their overall QOC as excellent by respondents’ demographics and characteristics of the provider and facility for their most recent care experience. Then, we used multivariable Poisson regression models with robust variance, as previously used in cross-sectional studies with high-prevalence dichotomized outcomes, to calculate adjusted prevalence ratios (aPRs) for predictors of excellent QOC [15]. Five multivariable models were calculated with four models using the individual domains of the responsiveness index as the exposure of interest and the fifth model using the summative responsiveness index. Using a ‘top-box’ comparison, we determined the aPR of excellent QOC vs not excellent (very good, good, fair and poor) for respondents in the highest quintile of the responsiveness index compared to those in the lower quintiles. Multivariable models were adjusted for country, age, gender, level of education, urban/rural-dwelling, person for whom care was sought, type of provider seen, public or private sector status of the facility and type of facility.

Analyses were performed using SAS 9.4 (SAS Institute Inc). We report two-sided 95% CIs for aPRs using *a* = 0.05 to indicate statistical significance for all comparisons.

## Results

Overall, there were 14 008 respondents who completed the survey, ranging from 2519 people in Kenya to 5658 people in India (Supplementary Material 2). Survey respondents tended to be young (89.4% younger than age 45), male (72.0%), well-educated (57.4% with a post-secondary degree) and urban-dwelling (65.7%) (Table 1). About a third of the respondents reported being on a prepayment program such as medical aid, insurance or a similar program.

**Table 1:** Demographics of survey respondents

	**All** **c** **ountries (*n*** ** ** **= 14 008)**	**India (*n*** ** ** **= 5** **658)**	**Kenya (*n*** ** ** **= 2** **519)**	**Mexico (*n*** ** ** **= 2** **934)**	**Nigeria (*n*** ** ** **= 2** **897)**
Age in years, no. (%)					
Under 25	6127 (43.7)	2467 (43.6)	1278 (50.7)	974 (33.2)	1408 (48.6)
25–44	6404 (45.7)	2723 (48.1)	1047 (41.6)	1314 (44.8)	1320 (45.6)
45–64	1112 (7.9)	348 (6.2)	139 (5.5)	492 (16.8)	133 (4.6)
65 and over	365 (2.6)	120 (2.1)	55 (2.2)	154 (5.3)	36 (1.2)
Female, no. (%)	3920 (28.0)	1442 (25.5)	598 (23.8)	1104 (37.6)	776 (26.8)
Education, no. (%)					
No school	806 (5.8)	426 (7.5)	156 (6.2)	144 (4.9)	80 (2.8)
Primary	523 (3.7)	188 (3.3)	172 (6.8)	128 (4.4)	35 (1.2)
Secondary	2980 (21.3)	884 (15.6)	824 (32.7)	457 (15.6)	815 (28.1)
Vocational	1654 (11.8)	476 (8.4)	167 (6.6)	808 (27.5)	203 (7.0)
Post-secondary	8044 (57.4)	3684 (65.1)	1199 (47.6)	1397 (47.6)	1764 (60.9)
Urban, no. (%)	9199 (65.7)	3443 (60.9)	1570 (62.3)	2225 (75.8)	1961 (67.7)
Prepayment program, no. (%)	4673 (33.4)	1806 (31.9)	968 (38.4)	1043 (35.6)	856 (29.6)

Baseline characteristics of all survey respondents, regardless of whether they received care in the prior 6 months, in order to demonstrate the demographics of the internet users sampled in the study.

Of the total respondents, 4191 people (29.9%) reported having sought care in the past 6 months, ranging from 19.9% of respondents in India to 40.5% in Kenya (Supplementary Material 3). People who sought care tended to be older, female, better educated, more likely to be urban-dwelling and more likely to be part of a prepayment program than those who did not seek care in the last 6 months. Meanwhile, those who did not seek care in the past 6 months reported not seeking care primarily due to not being sick (57.1%), other unspecified reasons (21.2%) or care being too expensive (10.0%) (Supplementary Material 4).

Among people who sought care in the prior 6 months, most sought care for themselves (46.1%) or another family member (28.5%) (Table 2). The most common reason for seeking care was an acute new problem or question such as fever, new diarrhea, rash, headache or worry about a new symptom or feeling (44.4%). Across all four countries, more people went to see a specialist physician (49.9%) than a primary care provider (26.1%), while most care was sought in a hospital (50.6%) rather than a primary care clinic (15.6%). The degree of care-seeking at public vs private sector facilities varied by country, with 68.0% of respondents in India reporting going to a private facility compared to 35.2% in Mexico. Additional care-seeking characteristics by reason for seeking care are presented in Supplementary Material 5.

**Table 2:** Characteristics of people who received care in the prior 6 months in India, Kenya, Mexico and Nigeria

	**All countries (*n* = 4191)**	**India (*n* = 1128)**	**Kenya (*n* = 1019)**	**Mexico (*n* = 1023)**	**Nigeria (*n* = 1021)**
Age in years, no. (%)					
Under 25	1595 (38.1)	441 (39.1)	466 (45.7)	285 (27.9)	403 (39.5)
25–44	2010 (48.0)	452 (40.1)	482 (47.3)	440 (43.0)	536 (52.5)
45–64	461 (11.0)	99 (8.8)	54 (5.3)	241 (23.6)	69 (6.8)
65 and over	125 (3.0)	36 (3.2)	19 (1.9)	57 (5.6)	13 (1.3)
Female, no. (%)	1394 (33.3)	374 (33.2)	263 (25.8)	444 (43.4)	313 (30.7)
Education, no. (%)					
No school	133 (3.2)	52 (4.6)	42 (4.1)	12 (1.2)	27 (2.6)
Primary	167 (4.0)	49 (4.3)	73 (7.2)	35 (3.4)	10 (1.0)
Secondary	942 (22.5)	177 (15.7)	346 (34.0)	158 (15.4)	263 (25.8)
Vocational	500 (11.9)	87 (7.7)	62 (6.1)	287 (28.1)	64 (6.3)
Post-secondary	2449 (48.4)	765 (67.8)	496 (48.7)	531 (51.9)	657 (64.3)
Urban, no. (%)	2935 (70.0)	750 (66.5)	651 (63.9)	830 (81.1)	704 (69.0)
Prepayment program, no. (%)	2007 (47.9)	592 (52.5)	485 (47.6)	509 (49.8)	421 (41.2)
Who received care, no. (%)					
Yourself	1932 (46.1)	546 (48.4)	453 (44.5)	438 (42.8)	495 (48.5)
Family member	1952 (46.6)	484 (42.9)	509 (50.0)	516 (50.4)	443 (43.4)
Other	306 (7.3)	98 (8.7)	56 (5.5)	69 (6.7)	83 (8.1)
Care reason, no. (%)					
Emergency care	453 (10.8)	130 (11.5)	95 (9.3)	142 (13.9)	86 (8.4)
Antenatal care	297 (7.1)	80 (7.1)	72 (7.1)	59 (5.8)	86 (8.4)
Childbirth	332 (7.9)	122 (10.8)	69 (6.8)	48 (4.7)	93 (9.1)
Routine care	890 (21.2)	248 (22.0)	167 (16.4)	294 (28.7)	181 (17.7)
Chronic existing problem	357 (8.5)	112 (10.0)	86 (8.4	114 (11.1)	45 (4.4)
Acute new problem	1862 (44.4)	436 (38.7)	530 (52.0)	366 (35.8)	530 (52.0)
Care provider, no. (%)					
Pharmacist or drug seller	446 (10.6)	103 (9.1)	103 (10.1)	88 (8.6)	152 (14.9)
CHW	558 (13.3)	144 (12.8)	108 (10.6)	189 (18.5)	117 (11.5)
Primary health-care provider	1095 (26.1)	302 (26.8)	272 (26.7)	283 (27.7)	238 (23.3)
Specialist provider	2092 (49.9)	579 (51.3)	536 (52.6)	463 (45.3)	514 (50.3)
Care location, no. (%)					
Drug store or pharmacy	294 (7.0)	48 (4.3)	58 (5.7)	82 (8.0)	106 (10.4)
Primary care clinic or office	653 (15.6)	167 (14.8)	117 (11.5)	206 (20.1)	163 (16.0)
Specialty clinic	450 (10.7)	140 (12.4)	54 (5.3)	190 (18.6)	66 (6.5)
Hospital or emergency room	2120 (50.6)	460 (40.8)	688 (67.5)	441 (43.1)	531 (51.9)
Home	410 (9.8)	221 (19.6)	59 (5.8)	48 (4.7)	82 (8.0)
Work or school	264 (6.3)	92 (8.2)	43 (4.2)	56 (5.5)	73 (7.2)
Facility type, no. (%)					
Public	1540 (43.8)	173 (21.2)	474 (51.7)	517 (56.3)	376 (43.4)
Private	1648 (46.9)	554 (68.0)	354 (38.6)	323 (35.2)	417 (48.2)
Faith-based organization	178 (5.1)	56 (6.9)	60 (6.5)	26 (2.8)	36 (4.2)
Don’t know	151 (4.3)	32 (3.9)	29 (3.2)	53 (5.8)	37 (4.3)

Baseline characteristics of people who reported having received medical care in the 6 months prior to the survey.

On a scale of 0–1, the mean responsiveness score was 0.66, with the highest average in Nigeria (mean 0.71, standard deviation 0.20) and the lowest in India and Mexico (both means 0.64, standard deviation 0.22) (Table 3). Of the responsiveness domains, the highest proportion of respondents gave the top rating for wait time (44.6%), while the lowest proportion gave the highest rating for facility cleanliness (21.7%).

**Table 3:** Health system responsiveness domains and QOC ratings by country

	**All c** **ountries (** ***n*** ** = 4** **191)**	**India (*n*** ** ** **= 1** **128)**	**Kenya (*n*** ** ** **= 1** **019)**	**Mexico (*n*** ** ** **= 1** **023)**	**Nigeria (*n*** ** ** **= 1** **021)**
Responsiveness index, mean (SD)	0.66 (0.22)	0.64 (0.22)	0.66 (0.22)	0.64 (0.22)	0.71 (0.20)
Highest rating of wait time, no. (%)	1870 (44.6)	529 (46.9)	438 (43.0)	409 (40.0)	494 (48.4)
Highest rating of facility cleanliness, no. (%)	764 (21.7)	152 (18.7)	213 (23.2)	152 (16.5)	247 (28.5)
Highest rating of understanding provider’s advice, no. (%)	1168 (27.9)	288 (25.5)	268 (26.3)	230 (22.5)	382 (37.4)
Highest rating of feeling respected by provider and staff, no. (%)	973 (23.2)	214 (19.0)	255 (25.0)	217 (21.2)	287 (28.1)
Highest rating of QOC, no. (%)	914 (21.8)	202 (17.9)	222 (21.8)	189 (18.5)	301 (29.5)

Mean scores on the responsiveness index, which was derived by re-scaling the ratings from the four domains (dignity/respect, quality of amenities/cleanliness, prompt attention/wait time, communication/provider’s advice) and calculating the average. Also presented are the proportions of the highest rating of the four responsiveness domains and patient-reported QOC.

Meanwhile, 21.8% of respondents gave the highest overall rating of QOC. For individual countries, India had the lowest proportion of excellent patient-reported QOC (17.9%), while Nigeria had the highest (29.5%). Internal consistency statistics of the survey questions using Likert scales demonstrated a standardized Cronbach’s coefficient alpha of 0.85, which was greater than the suggested value of 0.70 by Nunnally and Bernstein [16].

In an adjusted analysis, people experiencing the highest quintile of health system responsiveness were 8.6 times more likely to report excellent quality than those in lower quintiles (aPR 8.61, 95% CI: 7.50, 9.89; *P* < 0.01) (Table 4). Respondents aged 25–44 and 45–64 years were 15% and 23% less likely to report excellent QOC compared to those under age 25 years. Urban-dwelling individuals were less likely to report excellent quality compared to rural-dwelling individuals (aPR 0.88, 95% CI: 0.78, 0.99; *P* = 0.04). People who saw community health workers (CHWs; aPR 1.37, 95% CI: 1.12, 1.67; *P* < 0.01) and specialists (aPR 1.30, 95% CI: 1.12, 1.50; *P* < 0.01) were more likely to report excellent quality than those who saw primary care providers.

**Table 4:** Unadjusted and aPRs of excellent patient-reported QOC by health system responsiveness

	**Unadjusted prevalence ratio**	**95%** **CI**	***P*** **-value**	**aPR**	**95%** **CI**	***P*** **-value**
Highest quintile of responsiveness (ref: lower quintiles)	9.02	7.89, 10.3	<0.01	8.61	7.50, 9.89	<0.01
Country (ref: India)						
Kenya	1.29	1.06, 1.58	0.01	1.23	1.05, 1.45	0.01
Mexico	1.15	0.94, 1.42	0.17	1.21	1.04, 1.42	0.02
Nigeria	1.71	1.41, 2.06	<0.01	1.39	1.19, 1.62	<0.01
Age group (years, ref: under 25)						
25–44	0.81	0.70, 0.92	<0.01	0.85	0.76, 0.96	<0.01
45–64	0.73	0.58, 0.92	<0.01	0.77	0.65, 0.91	<0.01
65+	1.15	0.83, 1.60	0.39	1.03	0.79, 1.34	0.84
Female (ref: male)	0.98	0.86, 1,13	0.82	1.01	0.90, 1.12	0.91
Education (ref: no school)						
Primary	0.57	0.32, 0.99	0.047	0.94	0.58, 1.52	0.80
Secondary	0.94	0.64, 1,38	0.75	1.17	0.85, 1.62	0.34
Vocational	0.70	0.46, 1.05	0.09	0.98	0.69, 1.38	0.90
Post-secondary	0.75	0.51, 1.09	0.13	1.03	0.75, 1.41	0.87
Urban (ref: rural)	0.90	0.78, 1.03	0.13	0.88	0.78, 0.99	0.04
Prepayment plan (ref: no prepayment plan)	1.20	1.06, 1.36	<0.01	1.05	0.95, 1.16	0.36
Person receiving care (ref: yourself)						
Your child	0.72	0.60, 0.87	<0.01	0.91	0.78, 1.06	0.22
Another family member	0.81	0.69, 0.94	<0.01	0.92	0.81, 1.04	0.18
Other	0.95	0.73, 1.23	0.69	1.04	0.83, 1.29	0.74
Care reason (ref: routine care)						
Acute new problem	0.88	0.75, 1.04	0.13	0.90	0.79, 1.02	0.09
Antenatal care	0.89	0.67, 1.18	0.42	1.00	0.80, 1.25	0.99
Childbirth	1.09	0.84, 1.42	0.50	1.04	0.83, 1.29	0.75
Chronic existing problem	0.86	0.66, 1.12	0.27	1.09	0.87, 1,36	0.46
Emergency care	0.87	0.68, 1.11	0.25	0.92	0.75, 1.13	0.42
Provider type (ref: primary care)						
Pharmacist or drug seller	1.40	1.09, 1.80	0.01	1.08	0.86, 1.34	0.52
CHW	1.35	1.05, 1.74	0.02	1.37	1.12, 1.67	<0.01
Specialist	1.54	1.30, 1.83	<0.01	1.30	1.12, 1.50	<0.01
Care location (ref: primary care clinic)						
Drug store/pharmacy	1.19	0.92, 1.54	0.19	1.09	0.87, 1.35	0.46
Hospital	1.01	0.71, 1.44	0.95	1.17	0.88, 1.55	0.28
Specialty clinic	1.10	0.87, 1.39	0.41	0.97	0.81, 1.16	0.71
Private facility (ref: public)	1.30	1.14, 1.50	<0.01	1.09	0.97, 1.22	0.15

The unadjusted and aPRs of patients rating the quality of their care as excellent, as calculated by univariable and multivariable Poisson regression models. Using a ‘top-box’ comparison, we determined the prevalence ratio of excellent QOC vs not excellent (very good, good, fair, poor) for respondents in the highest quintile of the responsiveness index compared to those in the lower quintiles. The multivariable model was adjusted for country, age group, sex, education level, urban/rural status, prepayment plan, person who received care, reason for receiving care, type of provider seen, location of care and public/private status of the health facility.

Given this significant association between health system responsiveness and overall ratings of quality, Table 5 shows the unadjusted and adjusted relationship between the subcomponents of the responsiveness index and reported QOC. Perceived respect from provider or staff was most highly associated with excellent overall patient-reported QOC (aPR 14.05, 95% CI: 11.83, 16.70; *P* < 0.01), while rating of wait time corresponded the least (aPR 2.78, 95% CI: 2.42, 3.21).

**Table 5:** Association of responsiveness index and its domains with excellent patient-reported QOC

	**Unadjusted prevalence ratio**	**95% CI**	***P*-value**	**aPR**	**95% CI**	***P*-value**
Highest rating of wait time (ref: lower ratings of wait time)	2.77	2.44, 3.14	<0.01	2.78	2.42, 3.21	<0.01
Highest rating of cleanliness (ref: lower ratings of cleanliness)	6.85	6.03, 7.79	<0.01	6.43	5.62, 7.36	<0.01
Highest rating of understanding advice (ref: lower ratings of understanding advice)	6.76	5.95, 7.69	<0.01	6.54	5.63, 7.58	<0.01
Highest rating of respect from provider (ref: lower ratings of respect from provider)	13.87	11.96, 16.08	<0.01	14.05	11.83, 16.70	<0.01
Top quintile of responsiveness index (ref: lower quintiles of responsiveness index)	9.02	7.89, 10.30	<0.01	8.61	7.50, 9.89	<0.01

The unadjusted and aPRs of patients rating the quality of their care as excellent, as calculated by univariable and multivariable Poisson regression models. Five multivariable models were calculated with four models using the individual domains of the responsiveness index as the exposure of interest and the fifth model using the summative responsiveness index (full model detailed in Table 4). Using a ‘top-box’ comparison, we determined the prevalence ratio of excellent QOC vs not excellent (very good, good, fair, poor) for respondents in the highest quintile of the responsiveness index compared to those in the lower quintiles. The multivariable models were adjusted for country, age group, sex, education level, urban/rural status, prepayment plan, person who received care, reason for receiving care, type of provider seen, location of care and public/private status of the health facility.

## Discussion

### Statement of principal findings

In this survey of four LMICs, we found significant variations in people’s care-seeking behaviors, experiences and perceptions of the QOC. Only about a fifth of respondents reported experiencing the highest level of perceived QOC. People were more likely to report better care quality with specialists and CHWs compared to primary care providers, while rural-dwelling individuals reported better quality than people in urban areas. High health systems responsiveness was strongly associated with excellent ratings of QOC. Within the responsiveness index, respect from the provider and staff had the largest association with respondents’ overall quality rating.

### Interpretation within the context of the wider literature

Ratings of QOC in all four countries were poor with only about a fifth of respondents reporting excellent QOC. Meanwhile, in a study of primary care in the USA, 69–79% of respondents reported an excellent global rating of their health care depending on whether or not they received primary care [17]. In our survey, a low prevalence of excellent patient-reported quality is particularly concerning because we sampled internet users who were likely to represent the upper end of patient access, health status and experience [18]. As such, the population that was not surveyed due to lack of internet access may be disproportionately poorer and subsequently experience worse QOC, in terms of both user experience and competent care as highlighted by The Lancet Global Health Commission on High-Quality Health Systems in SDG era [1, 19].

In our analysis, India had the lowest prevalence of excellent patient-reported QOC while Nigeria had the highest. This mirrored a recent study of person-centeredness across 12 LMICs, which found that India had one of the lowest proportion of positive quality ratings and Nigeria had one of the highest [10]. There is limited national literature on person-centeredness in these four LMICs. A 2019 study in Kenya, Ghana and India found low levels of person-centered maternity care regardless of setting [20]. In India, a fifth of women giving childbirth reported mistreatment throughout pregnancy including discrimination and abuse [21]. A fifth of women in Kenya reported feeling humiliated during labor and delivery [22]. On the other hand, in Nigeria, studies at individual clinics and hospitals demonstrated relatively high levels of patient satisfaction with the QOC, which may be attributed to positive patient–provider relationships, communication and accessibility [23]. Although Nigeria generally shares similar health systems challenges with other LMICs, including insufficient human resources and underinvestment in public sector care, these studies suggest differences in the patient–provider relationship may have an outsized impact on perceptions of care.

Along those lines, we found that health system responsiveness and patients’ ratings of QOC were highly associated and that perceived respect from providers and staff was particularly important in how patients rated their overall care. This analysis resonates with our recent study of a nationally representative sample of women of reproductive age in Ghana [4]. Ghanaian women experiencing the highest levels of health system responsiveness were more likely to report excellent QOC as well as other outcomes such as self-reported health.

In our study, urban-dwelling individuals were less likely to report excellent quality compared to rural-dwelling individuals. One hypothesis for this difference is that people in rural areas experienced better respect from providers and staff, a major determinant of people’s ratings. In particular, these rural health-care workers may be CHWs, who were associated with higher patient-reported quality ratings in our study, with CHW programs more often implemented in rural areas in LMICs [24]. Another possibility is that patient experience and ratings may be influenced by people’s expectations of health-care quality. A 2017 survey in LMICs found that a majority gave a high rating of quality to vignettes that actually described poor-quality services, suggesting low expectations [11]. Although there was no significant difference between urban and rural-dwelling individuals in this study, it is possible that rural-dwelling individuals in our study countries had lower expectations of quality compared to urban-dwelling individuals and subsequently reported higher quality ratings.

We also noted that people rated their QOC higher when they received care from CHWs in comparison to primary care providers. A recent study in Mexico demonstrated that a CHW program may increase social connectedness [25]. Similarly, CHWs’ ability to bring better local, interpersonal relationships may contribute to this pattern of better patient satisfaction from care delivered by CHWs. As such, investments to improve the patient–provider relationship may advance person-centered care, patient retention and potentially patient adherence to care recommendations and outcomes [26].

Our study suggests that primary care providers across the four countries may underperform with regard to patient experience compared to other providers such as specialists and CHWs. There are few national studies in LMICs assessing patients’ experiences of care with different types of health-care providers. A cross-sectional study of patient satisfaction in Mexico demonstrated that patients seeing medical specialists had a 2.4 times greater odds of reporting positive patient satisfaction compared to general practitioners [27]. The reasons for this disparity are unclear from the literature, but it may reflect a general underinvestment in primary care—ranging from training and supervision to financial remuneration and support.

### Strengths and limitations

There were several strengths and limitations. First, the findings were limited to the English/Spanish-speaking, internet-using population of each country. Second, we did not adjust for multiple hypothesis testing as this was an exploratory and hypothesis-generating study. Third, due to the self-reporting nature of the survey, there was potential for recall and social desirability biases, However, internet surveys tend to minimize the social desirability bias [28]. In particular, the RDIT-based survey did not prompt or ask for any personally identifying information, so the risk of social desirability bias was likely lower than other methodologies that ask respondents to identify themselves. Fourth, because this was a cross-sectional survey, we could not account for any potential unmeasured confounders or draw causal relationships between our variables of interest. Fifth, the survey did not ask about patients’ health status, which has been shown to be an important predictor of patient experience. Finally, there was a relatively low response rate, which corresponded with other online surveys and was trade-off for the ability to collect many responses rapidly across four different countries [11]. Despite these limitations, there is a lack of global data on person-centeredness in LMICs, and our study utilized a novel methodology to rapidly survey thousands of people across four countries.

### Implications for policy, practice and research

Our findings provide avenues for further investigation in person-centeredness. The results highlight the need to test novel measures and innovative methods of data collection that harness technology for LMICs. Through advancing data collection and emphasizing person-centeredness, the global community of research, practice and policy can enhance its ability to better assess and improve the quality of health systems and care delivery around the world.

Meanwhile, health systems in LMICs may invest in interventions ranging from training and coaching to providing staffing support, more time spent communicating with the patient and better reimbursement for providing patient-centered care [29]. Given the evidence that increased health system expenditure is associated with higher responsiveness, targeted increases in investments, particularly in public and primary health care facilities, may be necessary to improve the experiential QOC [30].

## Conclusions

In a novel, internet-based survey of care-seeking behaviors and person-centeredness in four LMICs, we demonstrate that overall patient-reported QOC and health system responsiveness are low even among a well-educated, young population and vary according to characteristics that may highlight health inequities and disparities within countries. As LMICs seek to measure and improve the quality of their health-care systems, further research is necessary to elucidate differences in person-centeredness and utilize novel methodologies harnessing technology in order to achieve scale and rapid data collection. As countries strive to achieve universal health coverage, promoting person-centeredness will be crucial to ensure health systems that are truly high quality.

## Supplementary Material

mzab110_Supp

## Data Availability

The data underlying this article will be shared on reasonable request to the corresponding author.

## References

[1] Kruk ME , Gage AD, Arsenault C et al. High-quality health systems in the Sustainable Development Goals era: time for a revolution. *Lancet Glob Health* 2018;6:e1196–252. 30196093 10.1016/S2214-109X(18)30386-3 PMC7734391

[2] UN General Assembly . *Transforming our World: the 2030**A**genda for**S**ustainable**D**evelopment*. 2015 Oct. Report No.: A/RES/70/1. https://www.refworld.org/docid/57b6e3e44.html (26 Apr 2021, date last accessed).

[3] National Academies of Sciences, Engineering, and Medicine, Health and Medicine Division, Board on Health Care Services, Board on Global Health, Committee on Improving the Quality of Health Care Globally . *Crossing the Global Quality Chasm: Improving Health Care Worldwide*. Washington, DC: National Academies Press (US), 2018. 30605296

[4] Ratcliffe HL , Bell G, Awoonor-Williams K et al. Towards patient-centred care in Ghana: health system responsiveness, self-rated health and experiential quality in a nationally representative survey. *BMJ Open Qual* 2020;9:e000886. 10.1136/bmjoq-2019-000886 PMC7228562 32404309

[5] Browne K , Roseman D, Shaller D et al. Analysis & commentary. Measuring patient experience as a strategy for improving primary care. *Health Aff (Millwood)* 2010;29:921–5. 20439881 10.1377/hlthaff.2010.0238

[6] Doyle C , Lennox L, Bell D. A systematic review of evidence on the links between patient experience and clinical safety and effectiveness. *BMJ Open* 2013;3:e001570. 10.1136/bmjopen-2012-001570 PMC3549241 23293244

[7] Darby C , Valentine N, Murray CJ et al. World Health Organization (WHO): strategy on measuring responsiveness. Geneva, Switzerland: World Health Organization, 2003. Report No.: 23.

[8] Ilunga Kalenga O , Moeti M, Sparrow A et al. The ongoing Ebola epidemic in the Democratic Republic of Congo, 2018–2019. *N Engl J Med* 2019;381:373–83. 31141654 10.1056/NEJMsr1904253

[9] Freedman LP , Ramsey K, Abuya T et al. Defining disrespect and abuse of women in childbirth: a research, policy and rights agenda. *Bull World Health Organ* 2014;92:915–7. 25552776 10.2471/BLT.14.137869 PMC4264393

[10] Roder-dewan S , Gage A, Hirschhorn LR et al. Level of confidence in and endorsement of the health system among internet users in 12 low-income and middle-income countries. *BMJ Glob Health* 2020;5:e002205. 10.1136/bmjgh-2019-002205 PMC7454186 32859647

[11] Roder-dewan S , Gage AD, Hirschhorn LR et al. Expectations of healthcare quality: a cross-sectional study of internet users in 12 low- and middle-income countries. *PLoS Med* 2019;16:e1002879. 10.1371/journal.pmed.1002879 PMC6685603 31390364

[12] How It Works - RIWI . https://riwi.com/how-it-works/ (5 July 2020, date last accessed).

[13] Larson E , Sharma J, Bohren MA et al. When the patient is the expert: measuring patient experience and satisfaction with care. *Bull World Health Organ* 2019;97:563–9. 31384074 10.2471/BLT.18.225201 PMC6653815

[14] Internet Users by Country . *Internet**L**ive**S**tats*. 2016. https://www.internetlivestats.com/internet-users-by-country/ (20 April 2021, date last accessed).

[15] Guanais F , Doubova SV, Leslie HH et al. Patient-centered primary care and self-rated health in 6 Latin American and Caribbean countries: analysis of a public opinion cross-sectional survey. *PLoS Med* 2018;15:e1002673. 10.1371/journal.pmed.1002673 PMC6177127 30300422

[16] Nunnally JC , Bernstein IH. *Psychometric Theory*. 2nd edn New York: McGraw-Hill, 1994.

[17] Levine D , Landon B, Linder J. Quality and experience of outpatient care in the United States for adults with or without primary care. *JAMA Intern Med* 2019;179:363–72. 30688977 10.1001/jamainternmed.2018.6716 PMC6439688

[18] Couper MP , Miller PV. Web survey methods: introduction. *Public Opin Q* 2008;72:831–5.

[19] McKinsey . *Offline and Falling Behind:**B**arriers to**I**nternet Adoption*. https://www.mckinsey.com/industries/technology-media-and-telecommunications/our-insights/offline-and-falling-behind-barriers-to-internet-adoption (20 Jun 2021, date last accessed).

[20] Afulani PA , Phillips B, Aborigo RA et al. Person-centred maternity care in low-income and middle-income countries: analysis of data from Kenya, Ghana, and India. *Lancet Glob Health* 2019;7:e96–109. 30554766 10.1016/S2214-109X(18)30403-0 PMC6293963

[21] Raj A , Dey A, Boyce S et al. Associations between mistreatment by a provider during childbirth and maternal health complications in Uttar Pradesh, India. *Matern Child Health J* 2017;21:1821–33. 28676965 10.1007/s10995-017-2298-8

[22] Abuya T , Warren CE, Miller N et al. Exploring the prevalence of disrespect and abuse during childbirth in Kenya. *PLoS One* 2015;10:e0123606. 10.1371/journal.pone.0123606 PMC4401776 25884566

[23] Ogaji DS , Giles S, Daker-White G et al. Findings and predictors of patient-reported experience of primary health care in Nigeria. *J Patient Exp* 2016;3:69–80. 28725841 10.1177/2374373516667005 PMC5513649

[24] Wahl B , Lehtimaki S, Germann S et al. Expanding the use of community health workers in urban settings: a potential strategy for progress towards universal health coverage. *Health Policy Plan* 2020;35:91–101. 31651958 10.1093/heapol/czz133

[25] Deitz RL , Hellerstein LH, St George SM et al. A qualitative study of social connectedness and its relationship to community health programs in rural Chiapas, Mexico. *BMC Public Health* 2020;20:852. 10.1186/s12889-020-09008-6 PMC7271512 32493280

[26] Beach MC , Keruly J, Moore RD. Is the quality of the patient-provider relationship associated with better adherence and health outcomes for patients with HIV? *J Gen Intern Med* 2006;21:661–5. 16808754 10.1111/j.1525-1497.2006.00399.x PMC1924639

[27] Prado-Galbarro F-J , Cruz-Cruz C, Gamiño-Arroyo A-E et al. Satisfaction with healthcare services among patients with diabetes, hypertension, and/or dyslipidemia in Mexico: a cross-sectional study. *Value**Health Reg**Issues* 2020;23:19–24. 10.1016/j.vhri.2019.11.002 32062192

[28] Social Desirability . *SAGE**R**esearch**M**ethods*. https://methods.sagepub.com/reference/encyclopedia-of-survey-research-methods/n537.xml (5 July 2020, date last accessed).

[29] Murante AM , Seghieri C, Vainieri M et al. Patient-perceived responsiveness of primary care systems across Europe and the relationship with the health expenditure and remuneration systems of primary care doctors. *Soc Sci Med* 2017;186:139–47. 28647664 10.1016/j.socscimed.2017.06.005

[30] Malhotra C , Do YK. Public health expenditure and health system responsiveness for low-income individuals: results from 63 countries. *Health Policy Plan* 2017;32:314–9. 27651279 10.1093/heapol/czw127

