# Supplementary material for: Predictors of patient-reported quality of care in low- and middle-income countries: a four-country survey of person-centered care
Source: Int J Qual Health Care. 2021 Jul 28;33(3):mzab110. doi: 10.1093/intqhc/mzab110 (PMC8519224; doi:10.1093/intqhc/mzab110)
Supplement: mzab110_Supp [file mzab110_supp.zip › RIWI PRQOC Manuscript - Supplement.docx]

**“Predictors of Patient-Reported Quality of Care in Low and Middle-Income Countries: A Four-Country Internet Survey of Person-Centered Care”**

**Manuscript Ref. No.: INTQHC-2020-10-0788**

**Supplementary Material 1. Survey Questionnaire**

| # | **Question** | Answer Choices | Relevance |
| --- | --- | --- | --- |
|  | **How old were you at your last birthday?** | _______ | All |
|  | **What is the highest level of school you attended?** | No school 1  Primary 2  Secondary 3  Vocational 4  Post-secondary college or university 5 | All |
|  | **What is your gender?** | Female  Male | All |
|  | **What type of area do you live in?** | Urban 1  Rural 2 | All |
|  | **Have you received care for yourself or your child in the last six months that did not involve admission to a hospital?** | Yes 1  No 0 | All |
| *5b* | ***Why not?*** | *Not sick/did not need care 1/0*  *Facility is too far away 1/0*  *Too expensive 1/0*  *Too difficult to get to 1/0*  *Distrust of provider/facility 1/0*  *Negative prior experience 1/0*  *Lack of privacy or confidentiality 1/0*  *Did not know where to go 1/0*  *Other 1/0*  *Do not know -88* | *No care received in last 6 months* |
|  | **Who was the recipient of this care?** | Yourself 1  Your child 2  Another family member 3  Other 4 | Any care received in last 6 months |
|  | **Who did you see for this most recent care experience?** | Pharmacist or drug seller 1  Community health worker 2  Primary health care doctor/clinical officer/nurse 3  Specialist doctor/clinical officer/nurse 4 | Any care received in last 6 months |
|  | **If you went to a health facility, was it public or private?** | Public 1  Private 2  Do not know 3  NA 4 | Any care received in last 6 months |
|  | **Where did you receive care?** | Drug store or pharmacy 1  Primary care clinic or office 2  Hospital/ED 3  Other specialty clinic 4  Home 5 | Any care received in last 6 months |
|  | **How long did it take you to get to this location?** | 1-14 minutes 1  15-29 minutes 2  30-59 minutes 3  1-2 hours 4  more than 2 hours 5 | Any care received in last 6 months |
|  | **For what reasons were you seeking care?** | Emergency care, for example trauma 1  Antenatal care 2  Childbirth 3  Routine care, for example immunizations, family planning, or growth monitoring 4  Chronic existing problem, for example HIV, TB, diabetes 5  Acute new problem or question, for example fever, new diarrhea, rash, headache, worried about a new symptom or feeling 6 | Any care received in last 6 months |
|  | **How long did you wait before being seen by your provider?** | X minutes 1  X hours 2  Gave up without seeing provider 3 | Any care received in last 6 months |
|  | **How would you rate this waiting time?** | Unbearable 1  Very long 2  Long 3  A little long 4  Fine 5 | Any care received in last 6 months |
|  | **How would you rate the cleanliness of the rooms inside the facility, including toilets?** | Excellent 1  Very good 2  Good 3  Fair 4  Poor 5 | Any care received in last 6 months |
|  | **Do you agree or disagree with the following statement: You trust the skills and abilities of the health workers you saw.** | Strongly agree 1  Agree 2  Disagree 3  Strongly disagree 4 | Any care received in last 6 months |
|  | **How easy or difficult was it to understand the information your provider gave to you?** | Very difficult 1  Difficult 2  Easy 3  Very easy 4 | Any care received in last 6 months |
|  | **How easy or difficult was it for you to follow the provider’s advice?** | Very difficult 1  Difficult 2  Easy 3  Very easy 4 | Any care received in last 6 months |
|  | **How would you rate the level of respect shown to you by the providers and staff during this visit?** | Excellent 1  Very Good 2  Good 3  Fair 4  Poor 5 | Any care received in last 6 months |
|  | **Overall, taking everything into account, how would you rate the quality of care you received?** | Excellent 1  Very Good 2  Good 3  Fair 4  Poor 5 | Any care received in last 6 months |
|  | **How likely are you to return or bring your children to this facility/seek care from this same provider in the future?** | Extremely likely 1  Likely 2  Unlikely 3  Extremely unlikely 4 | Any care received in last 6 months |
|  | **How easy or difficult was it for you to pay for this visit?** | Very difficult 1  Difficult 2  Easy 3  Very easy 4 | Any care received in last 6 months |
|  | **Did you have to borrow money or sell something to afford the costs of this visit, including all costs such as transportation and lost wages?** | Yes 1  No 2 | Any care received in last 6 months |
|  | **Do you agree or disagree with the following statement: I had confidence in the health worker(s) who looked after me.** | Strongly agree 1  Agree 2  Disagree 3  Strongly disagree 4 | Any care received in last 6 months |
|  | **Are you a part of any prepayment plan (such as medical aid, insurance or a similar program) or institutional arrangement that pays for some or all of the health care services you receive?** | Yes 1  No 2 | All |
|  | **If you or your child were to get sick tomorrow, how easy or difficult would it be for you to get the care you need?** | Very difficult 1  Difficult 2  Easy 3  Very easy 4 | All |

**Supplementary Material 2. CONSORT Flow Diagram**

**All Countries**

Total Surveyed

(n = 61,987)

Total reporting **seeking no care** in the past 6 months

(n = 18,109)

Total reporting **seeking care** in the past 6 months

(n = 17,441)

Total completing survey

(n = 9,816)

Total completing survey

(n = 4,191)

**India**

Total Surveyed

(n = 33,875)

Total reporting **seeking no care** in the past 6 months

(n = 9,607)

Total reporting **seeking care** in the past 6 months

(n = 7,411)

Total completing survey

(n = 4,530)

Total completing survey

(n = 1,128)

**Kenya**

Total Surveyed

(n = 8,065)

Total reporting **seeking no care** in the past 6 months

(n = 2,391)

Total reporting **seeking care** in the past 6 months

(n = 3,131)

Total completing survey

(n = 1,499)

Total completing survey

(n = 1,019)

**Mexico**

Total Surveyed

(n = 9,934)

Total reporting **seeking no care** in the past 6 months

(n = 3,280)

Total reporting **seeking care** in the past 6 months

(n = 2,920)

Total completing survey

(n = 1,911)

Total completing survey

(n = 1,023)

**Nigeria**

Total Surveyed

(n = 10,113)

Total reporting **seeking no care** in the past 6 months

(n = 2,831)

Total reporting **seeking care** in the past 6 months

(n = 3,979)

Total completing survey

(n = 1,876)

Total completing survey

(n = 1,021)

**Supplementary Material 3. Demographics of People Who Did and Did Not Seek Care in the Past Six Months.**

|  | **Sought care (N=4,191)** | **Did not seek care (N=9,816)** |
| --- | --- | --- |
| **Countries, no. (%)**  **India**  **Kenya**  **Mexico**  **Nigeria** | 1,128 (19.9)  1,019 (40.5)  1,023 (34.9)  1,021 (35.2) | 4,530 (80.1)  1,499 (59.5)  1,911 (65.1)  1,876 (64.8) |
| **Age in yrs, no. (%)**  **Under 25**  **25-44**  **45-64**  **65 and over** | 1,595 (26.0)  2,010 (31.4)  461 (41.5)  125 (34.3) | 4,532 (74.0)  4,393 (68.6)  651 (58.5)  240 (65.8) |
| **Gender, no. (%)**  **Female**  **Male** | 1,394 (35.6)  2,797 (27.7) | 2,525 (64.4)  7,290 (72.3) |
| **Education, no. (%)**  **No school**  **Primary**  **Secondary**  **Vocational**  **Post-secondary** | 133 (16.5)  167 (31.9)  9422 (31.6)  500 (30.2)  2,449 (30.5) | 673 (83.5)  356 (68.1)  2,038 (68.4)  1,154 (69.8)  5,594 (69.6) |
| **Urban/rural, no . (%)**  **Urban**  **Rural** | 2,935 (31.9)  1,256 (26.1) | 6,263 (68.1)  3,553 (73.9) |
| **Prepayment program, no. (%)**  **Yes**  **No** | 2,007 (43.0)  2,184 (23.4) | 2,666 (57.1)  7,149 (76.6) |

**Supplementary Material 4. No Care-Seeking in the Past Six Months**

|  | **All Countries** | **India** | **Kenya** | **Mexico** | **Nigeria** |
| --- | --- | --- | --- | --- | --- |
| **Did not seek care in the past 6 months** | 9,816 | 4,530 | 1,499 | 1,911 | 1,876 |
| **Why did you not seek care?**  **Not sick**  **Too difficult to get to**  **Too expensive**  **Distrust of provider**  **Negative prior experience**  **Lack of privacy**  **Other** | 5,603 (57.1)  368 (3.8)  982 (10.0)  212 (2.2)  229 (2.3)  337 (3.4)  2,085 (21.2) | 2,422 (53.5)  123 (2.7)  349 (7.7)  90 (2.0)  91 (2.0)  173 (3.8)  1,282 (28.3) | 875 (58.4)  92 (6.1)  226 (15.1)  33 (2.2)  38 (2.5)  54 (3.6)  181 (12.1) | 1,070 (56.0)  65 (3.4)  185 (9.7)  51 (2.7)  53 (2.8)  60 (3.1)  427 (22.3) | 1,236 (65.9)  88 (4.7)  222 (11.8)  38 (2.0)  47 (2.5)  50 (2.7)  195 (10.4) |

**Supplementary Material 5. Care Reason, Type of Provider, Location, and Facility Type**

|  | **Pharmacist or drug seller (N=446)** | **Community health worker (N=558)** | **Primary care (N=1,095)** | **Specialist (N=2,092)** |
| --- | --- | --- | --- | --- |
| **Reason for seeking care, no. (%)**  **Emergency care**  **Antenatal care**  **Childbirth**  **Routine care**  **Chronic existing problem**  **Acute new problem** | 52 (11.7)  34 (7.6)  44 (9.9)  83 (18.6)  28 (6.3)  205 (46.0) | 65 (11.7)  55 (9.9)  74 (13.3)  131 (23.5)  51 (9.1)  182 (32.6) | 99 (9.0)  71 (6.5)  76 (6.9)  240 (21.9)  83 (7.6)  526 (48.0) | 237 (11.3)  137 (6.6)  138 (6.6)  436 (20.8)  195 (9.3)  949 (45.4) |

|  | **Primary care clinic (N=653)** | **Specialty clinic (N=450)** | **Hospital (N=2,120)** | **Drug store or pharmacy (N=294)** | **Home (N=410)** | **Work/school (N=264)** |
| --- | --- | --- | --- | --- | --- | --- |
| **Reason for seeking care, no. (%)**  **Emergency care**  **Antenatal care**  **Childbirth**  **Routine care**  **Chronic existing problem**  **Acute new problem** | 37 (5.7)  32 (4.9)  42 (6.4)  190 (29.1)  64 (9.8)  288 (44.1) | 39 (8.7)  26 (5.8)  26 (5.8)  115 (25.6)  50 (11.1)  194 (43.1) | 279 (13.2)  162 (7.6)  153 (7.2)  364 (17.2)  172 (8.1)  990 (46.7) | 17 (5.8)  15 (5.1)  10 (3.4)  54 (18.4)  17 (5.8)  181 (61.6) | 45 (11.0)  41 (10.0)  65 (15.6)  98 (23.9)  38 (9.3)  123 (30.0) | 36 (13.6)  21 (8.0)  35 (13.6)  69 (26.1)  16 (6.1)  86 (32.6) |

|  | **Public (N=1,540)** | **Private (N=1,648)** | **Faith-based organization (N=178)** | **Don’t know (N=151)** |
| --- | --- | --- | --- | --- |
| **Reason for seeking care, no. (%)**  **Emergency care**  **Antenatal care**  **Childbirth**  **Routine care**  **Chronic existing problem**  **Acute new problem** | 170 (11.0)  103 (6.7)  97 (6.3)  335 (21.8)  151 (9.8)  684 (44.4) | 160 (9.7)  100 (6.1)  96 (5.8)  328 (19.9)  116 (7.0)  848 (51.5) | 21 (11.8)  23 (12.9)  23 (12.9)  32 (18.0)  19 (10.7)  60 (33.7) | 21 (13.9)  9 (6.0)  15 (10.0)  28 (18.5)  17 (11.3)  61 (40.4) |

**Supplementary Table 5. Characteristics of Excellent and Not Excellent QOC**

|  | **All Countries (N=4,191)** | | **India (N=1,128)** | | **Kenya (N=1,019)** | | **Mexico (N=1,023)** | | **Nigeria (N=1,021)** | |
| --- | --- | --- | --- | --- | --- | --- | --- | --- | --- | --- |
|  | **Excellent QOC (N=914)** | **Not Excellent QOC (N=3,277)** | **Excellent QOC (N=202)** | **Not Excellent QOC (N=926)** | **Excellent QOC (N=222)** | **Not Excellent QOC (N=797)** | **Excellent QOC (N=189)** | **Not Excellent QOC (N=834)** | **Excellent QOC (N=301)** | **Not Excellent QOC (N=720)** |
| **Age in yrs, no. (%)**  **Under 25**  **25-44**  **45-64**  **65 and over** | 399 (43.7)  401 (43.9)  82 (9.0)  32 (3.5) | 1,196 (36.5)  1,609 (49.1)  379 (11.6)  93 (2.8) | 82 (40.6)  98 (48.5)  15 (7.4)  7 (3.5) | 359 (38.8)  454 (49.0)  84 (9.1)  29 (3.1) | 115 (51.8)  89 (40.1)  9 (4.1)  9 (4.1) | 351 (44.0)  393 (49.3)  43 (5.4)  10 (1.3) | 61 (32.3)  75 (39.7)  43 (22.8)  10 (5.3) | 224 (26.9)  365 (43.8)  198 (23.7)  47 (5.6) | 141 (46.8)  139 (46.2)  15 (5.0)  6 (2.0) | 262 (36.4)  397 (55.1)  54 (7.5)  7 (1.0) |
| **Female, no. (%)** | 297 (32.5) | 1,097 (33.5) | 62 (30.7) | 312 (33.7) | 56 (25.2) | 207 (26.0) | 99 (32.9) | 214 (29.7) | 99 (32.9) | 214 (29.7) |
| **Education, no. (%)**  **No school**  **Primary**  **Secondary**  **Vocational**  **Post-secondary** | 37 (4.1)  29 (3.2)  240 (26.3)  95 (10.4)  513 (56.1) | 96 (2.9)  138 (4.2)  702 (21.4)  405 (12.4)  1,936 (59.1) | 12 (5.9)  6 (3.0)  36 (17.8)  14 (6.9)  134 (66.3) | 40 (4.3)  43 (4.6)  139 (15.0)  73 (7.9)  631 (68.1) | 12 (5.4)  14 (6.3)  82 (37.0)  17 (7.7)  97 (43.7) | 30 (3.8)  59 (7.4)  264 (33.1)  45 (5.7)  399 (50.1) | 2 (1.1)  3 (1.6)  23 (12.2)  45 (23.8)  116 (61.4) | 10 (1.2)  32 (3.8)  135 (16.2)  242 (29.0)  415 (49.8) | 11 (3.7)  6 (2.0)  99 (32.9)  19 (6.3)  166 (55.2) | 16 (2.2)  4 (0.6)  164 (22.8)  45 (6.3)  491 (68.2) |
| **Urban, no. (%)** | 620 (67.8) | 2,315 (70.6) | 124 (61.4) | 626 (67.6) | 142 (64.0) | 509 (63.9) | 154 (81.5) | 676 (81.1) | 200 (66.5) | 504 (70.0) |
| **Prepayment program, no. (%)** | 480 (52.5) | 1,527 (46.6) | 118 (58.4) | 474 (51.2) | 118 (53.2) | 367 (46.1) | 107 (56.6) | 402 (48.2) | 137 (45.5) | 284 (39.4) |
| **Who Received Care, no. (%)**  **Yourself**  **Your child**  **Another family member**  **Other** | 471 (51.5)  129 (14.1)  246 (26.9)  68 (7.4) | 1,461 (44.6)  628 (19.2)  949 (29.0)  238 (7.3) | 109 (54.0)  16 (7.9)  55 (27.2)  22 (10.9) | 437 (47.2)  127 (13.7)  286 (30.9)  76 (8.2) | 116 (52.3)  33 (14.9)  61 (27.5)  12 (5.4) | 337 (42.3)  170 (21.4)  245 (30.8)  44 (5.5) | 89 (47.1)  44 (23.3)  44 (23.3)  12 (6.4) | 349 (41.9)  205 (24.6)  223 (26.7)  57 (6.8) | 157 (52.2)  36 (12.0)  86 (28.6)  22 (7.3) | 338 (47.0)  126 (17.5)  195 (27.1)  61 (8.5) |
| **Care Reason, no. (%)**  **Emergency care**  **Antenatal care**  **Childbirth**  **Routine care**  **Chronic existing problem**  **Acute new problem** | 92 (10.1)  62 (6.8)  86 (9.4)  205 (22.4)  78 (8.5)  391 (42.8) | 361 (11.0)  235 (7.2)  246 (7.5)  685 (20.9)  279 (8.5)  1,471 (44.9) | 25 (12.4)  15 (7.4)  20 (9.9)  54 (26.7)  28 (13.9)  60 (29.7) | 105 (11.3)  65 (7.0)  102 (11.0)  194 (21.0)  84 (9.1)  376 (40.6) | 19 (8.6)  13 (5.9)  25 (11.3)  35 (15.8)  25 (11.3)  105 (47.3) | 76 (9.5)  59 (7.4)  44 (5.5)  132 (16.6)  61 (7.7)  425 (53.3) | 26 (13.8)  11 (5.8)  6 (3.2)  63 (33.3)  11 (5.8)  72 (38.1) | 116 (13.9)  48 (5.8)  42 (5.0)  231 (27.7)  103 (12.4)  294 (35.3) | 22 (7.3)  23 (7.6)  35 (11.6)  53 (17.6)  14 (4.7)  154 (51.2) | 64 (8.9)  63 (8.8)  58 (8.1)  128 (17.8)  31 (4.3)  376 (52.2) |
| **Care Provider, no. (%)**  **Pharmacist/drug seller**  **Community health worker**  **Primary care provider**  **Specialist** | 98 (10.7)  118 (12.9)  187 (20.5)  511 (55.9) | 348 (10.6)  440 (13.4)  908 (27.7)  1,581 (48.3) | 21 (10.4)  31 (15.4)  38 (18.8)  112 (55.5) | 82 (8.9)  113 (12.2)  264 (28.5)  467 (50.4) | 17 (7.7)  32 (14.4)  44 (19.8)  129 (58.1) | 86 (10.8)  76 (9.5)  228 (28.6)  407 (51.1) | 14 (7.4)  15 (7.9)  45 (23.8)  115 (60.9) | 74 (8.9)  174 (20.9)  238 (28.5)  348 (41.7) | 46 (15.3)  40 (13.3)  60 (19.9)  155 (51.5) | 106 (14.7)  77 (10.7)  178 (24.7)  359 (49.9) |
| **Care Location, no. (%)**  **Drug store/pharmacy**  **Primary care clinic/office**  **Specialty clinic**  **Hospital**  **Home**  **Work/School** | 69 (7.6)  129 (14.1)  98 (10.7)  444 (48.6)  99 (10.8)  75 (8.2) | 225 (6.9)  524 (16.0)  352 (10.7)  1,676 (51.1)  311 (9.5)  189 (5.8) | 12 (5.9)  25 (12.4)  25 (12.4)  71 (35.1)  46 (22.8)  23 (11.4) | 36 (3.9)  142 (15.3)  115 (12.4)  389 (42.0)  175 (18.9)  69 (7.5) | 11 (5.0)  24 (10.8)  11 (5.0)  147 (66.2)  18 (8.1)  11 (5.0) | 57 (5.9)  93 (11.7)  43 (5.4)  541 (67.9)  41 (5.1)  32 (4.0) | 15 (7.9)  33 (17.5)  46 (24.3)  79 (41.8)  5 (2.7)  11 (5.8) | 67 (8.0)  173 (20.7)  144 (17.3)  362 (43.4)  43 (5.2)  45 (5.4) | 31 (10.3)  47 (15.6)  16 (5.3)  147 (48.8)  30 (10.0)  30 (10.0) | 75 (10.4)  116 (16.1)  50 (6.9)  374 (51.9)  52 (7.2)  43 (6.0) |
| **Facility Type, no. (%)**  **Public**  **Private**  **Faith-based organization**  **Don’t know** | 278 (37.6)  388 (52.4)  40 (5.4)  34 (4.6) | 1,262 (45.4)  1,260 (45.4)  138 (5.0)  117 (4.2) | 30 (22.6)  88 (66.2)  7 (5.3)  8 (6.0) | 143 (21.0)  466 (68.3)  49 (7.2)  24 (3.5) | 99 (51.3)  66 (34.2)  19 (9.8)  9 (4.7) | 375 (51.8)  288 (39.8)  41 (5.7)  20 (2.8) | 61 (35.3)  106 (61.3)  0 (0.0)  6 (3.5) | 456 (61.1)  217 (29.1)  26 (3.5)  47 (6.3) | 88 (36.5)  128 (53.1)  14 (5.8)  11 (4.6) | 288 (46.1)  289 (46.2)  22 (3.5)  26 (4.2) |

**Supplementary Table 6. Other Survey Results of People Who Sought Care (Wait time, Affordability, Returning to Facility, etc)**

|  | **All Countries (N=4,191)** | **India (N=1,128)** | **Kenya (N=1,019)** | **Mexico (N=1,023)** | **Nigeria (N=1,021)** |
| --- | --- | --- | --- | --- | --- |
| **Difficulty paying for visit, no. (%)**  **Very difficult**  **Difficult**  **Easy**  **Very easy**  **Did not pay** | 324 (7.7)  1,147 (27.4)  1,540 (36.8)  543 (13.0)  637 (15.2) | 96 (8.5)  260 (23.1)  460 (40.8)  156 (13.8)  156 (13.8) | 114 (11.2)  397 (39.0)  286 (28.1)  113 (11.1)  109 (10.7) | 51 (5.0)  231 (22.6)  350 (34.2)  97 (9.5)  294 (28.7) | 63 (6.2)  259 (25.4)  444 (43.5)  177 (17.3)  78 (7.6) |
| **Borrowed or sold items to afford visit, no. (%)**  **Yes**  **No** | 1,607 (38.3)  2,584 (61.7) | 442 (39.2)  686 (60.8) | 549 (53.9)  470 (46.1) | 258 (25.2)  765 (74.8) | 358 (35.1)  663 (64.9) |
| **Wait time, no. (%)**  **Gave up without seeing provider**  **Over 4 hrs**  **3-4 hrs**  **1-2 hrs**  **30-59 min**  **1-29 min** | 149 (3.6)  162 (3.9)  178 (4.3)  656 (15.7)  926 (22.1)  2,120 (50.6) | 82 (7.3)  48 (4.3)  38 (3.4)  170 (15.1)  207 (18.4)  583 (51.7) | 20 (2.0)  52 (5.1)  34 (3.3)  133 (13.1)  253 (24.8)  527 (51.7) | 31 (3.0)  32 (3.1)  64 (6.3)  197 (19.3)  222 (21.7)  477 (46.6) | 16 (1.6)  30 (2.9)  42 (4.1)  156 (15.3)  244 (23.9)  533 (52.2) |
| **Trust in health worker, no. (%)**  **Strongly disagree**  **Disagree**  **Agree**  **Strongly agree** | 179 (4.3)  369 (8.8)  2,665 (63.6)  978 (23.3) | 73 (6.5)  117 (10.4)  703 (62.3)  235 (20.8) | 26 (2.6)  71 (7.0)  694 (68.1)  228 (22.4) | 53 (5.2)  146 (14.3)  576 (56.3)  248 (24.2) | 27 (2.6)  35 (3.4)  692 (67.8)  267 (26.2) |
| **Confidence in health worker, no. (%)**  **Strongly disagree**  **Disagree**  **Agree**  **Strongly agree** | 164 (3.9)  458 (10.9)  2,660 (63.5)  909 (21.7) | 65 (5.8)  138 (12.2)  709 (62.9)  216 (19.2) | 30 (2.9)  89 (8.7)  678 (66.5)  222 (21.8) | 36 (3.5)  158 (15.4)  618 (60.4)  211 (20.6) | 33 (3.2)  73 (7.2)  655 (64.2)  260 (25.5) |
| **Difficulty following advice, no. (%)**  **Very difficult**  **Difficult**  **Easy**  **Very easy** | 136 (3.3)  446 (10.6)  2,453 (58.5)  1,156 (27.6) | 61 (5.4)  167 (14.8)  643 (57.0)  257 (22.8) | 23 (2.3)  99 (9.7)  604 (59.3)  293 (28.8) | 29 (2.8)  129 (12.6)  628 (61.4)  237 (23.2) | 23 (2.3)  51 (5.0)  578 (56.6)  369 (36.1) |
| **Likelihood of returning to provider, no. (%)**  **Extremely unlikely**  **Unlikely**  **Likely**  **Extremely likely** | 55 (8.2)  89 (13.2)  351 (52.1)  179 (26.6) | 32 (10.2)  36 (11.5)  166 (53.0)  79 (25.2) | 4 (3.9)  14 (13.7)  60 (58.8)  24 (23.5) | 13 (12.5)  24 (23.1)  47 (45.2)  20 (19.2) | 6 (3.9)  15 (9.7)  78 (50.3)  56 (36.1) |
| **Likelihood of returning to facility, no. (%)**  **Extremely unlikely**  **Unlikely**  **Likely**  **Extremely likely** | 121 (3.4)  337 (9.6)  2,082 (59.2)  977 (27.8) | 33 (4.1)  94 (11.5)  512 (62.8)  176 (21.6) | 27 (2.9)  81 (8.8)  558 (60.9)  251 (27.4) | 36 (3.9)  99 (10.8)  492 (53.5)  292 (31.8) | 25 (2.9)  63 (7.3)  520 (60.1)  258 (29.8) |

**Supplementary Table 7. STROBE Statement—Checklist of items that should be included in reports of *cross-sectional studies***

|  | **Item No** | **Recommendation** | **Page No** |
| --- | --- | --- | --- |
| **Title and abstract** | 1 | (*a*) Indicate the study’s design with a commonly used term in the title or the abstract | 1 |
|  |  | (*b*) Provide in the abstract an informative and balanced summary of what was done and what was found | 1 |
| **Introduction** | | | |
| Background/rationale | 2 | Explain the scientific background and rationale for the investigation being reported | 3 |
| Objectives | 3 | State specific objectives, including any prespecified hypotheses | 4 |
| **Methods** | | | |
| Study design | 4 | Present key elements of study design early in the paper | 4 |
| Setting | 5 | Describe the setting, locations, and relevant dates, including periods of recruitment, exposure, follow-up, and data collection | 5 |
| Participants | 6 | (*a*) Give the eligibility criteria, and the sources and methods of selection of participants | 5 |
| Variables | 7 | Clearly define all outcomes, exposures, predictors, potential confounders, and effect modifiers. Give diagnostic criteria, if applicable | 5 |
| Data sources/ measurement | 8* | For each variable of interest, give sources of data and details of methods of assessment (measurement). Describe comparability of assessment methods if there is more than one group | 5 |
| Bias | 9 | Describe any efforts to address potential sources of bias | 6 |
| Study size | 10 | Explain how the study size was arrived at | 5 |
| Quantitative variables | 11 | Explain how quantitative variables were handled in the analyses. If applicable, describe which groupings were chosen and why | 6 |
| Statistical methods | 12 | (*a*) Describe all statistical methods, including those used to control for confounding | 6 |
|  |  | (*b*) Describe any methods used to examine subgroups and interactions | -- |
|  |  | (*c*) Explain how missing data were addressed | -- |
|  |  | (*d*) If applicable, describe analytical methods taking account of sampling strategy | -- |
|  |  | (*e*) Describe any sensitivity analyses | -- |
| **Results** | | | |
| Participants | 13* | (a) Report numbers of individuals at each stage of study—eg numbers potentially eligible, examined for eligibility, confirmed eligible, included in the study, completing follow-up, and analysed | 7 |
|  |  | (b) Give reasons for non-participation at each stage | 7 |
|  |  | (c) Consider use of a flow diagram | Supp 2 |
| Descriptive data | 14* | (a) Give characteristics of study participants (eg demographic, clinical, social) and information on exposures and potential confounders | 7 |
|  |  | (b) Indicate number of participants with missing data for each variable of interest | -- |
| Outcome data | 15* | Report numbers of outcome events or summary measures | 8 |
| Main results | 16 | (*a*) Give unadjusted estimates and, if applicable, confounder-adjusted estimates and their precision (eg, 95% confidence interval). Make clear which confounders were adjusted for and why they were included | 8 |
|  |  | (*b*) Report category boundaries when continuous variables were categorized | 7 |
|  |  | (*c*) If relevant, consider translating estimates of relative risk into absolute risk for a meaningful time period | -- |
| Other analyses | 17 | Report other analyses done—eg analyses of subgroups and interactions, and sensitivity analyses | -- |
| **Discussion** | | | |
| Key results | 18 | Summarise key results with reference to study objectives | 9 |
| Limitations | 19 | Discuss limitations of the study, taking into account sources of potential bias or imprecision. Discuss both direction and magnitude of any potential bias | 11 |
| Interpretation | 20 | Give a cautious overall interpretation of results considering objectives, limitations, multiplicity of analyses, results from similar studies, and other relevant evidence | 9 |
| Generalisability | 21 | Discuss the generalisability (external validity) of the study results | 11 |
| **Other information** | | | |
| Funding | 22 | Give the source of funding and the role of the funders for the present study and, if applicable, for the original study on which the present article is based | 13 |

*Give information separately for exposed and unexposed groups.

**Note:** An Explanation and Elaboration article discusses each checklist item and gives methodological background and published examples of transparent reporting. The STROBE checklist is best used in conjunction with this article (freely available on the Web sites of PLoS Medicine at http://www.plosmedicine.org/, Annals of Internal Medicine at http://www.annals.org/, and Epidemiology at http://www.epidem.com/). Information on the STROBE Initiative is available at www.strobe-statement.org.
